# A polysaccharide from *Dendrobium huoshanense* prevents hepatic inflammatory response caused by carbon tetrachloride

**DOI:** 10.1080/13102818.2014.987514

**Published:** 2014-12-06

**Authors:** Chang-Cheng Tian, Xue-Qiang Zha, Jian-Ping Luo

**Affiliations:** ^a^ Hefei University of Technology, School of Biotechnology and Food Engineering, Hefei, China; ^b^ Bengbu College, Department of Biotechnology and Food Engineering, Bengbu, China

**Keywords:** *Dendrobium huoshanense*, polysaccharide, hepatoprotection, anti-inflammation

## Abstract

*Dendrobium huoshanense* is a precious herbal medicine in China, which exhibits a variety of restorative and therapeutic effects. This study aimed at investigating the hepatoprotective effects of a polysaccharide (DHP1A) isolated from *D. huoshanense* via water extraction, diethylaminoethyl (DEAE) cellulose anion exchange and size exclusion chromatography. The animal experiment indicated that the oral administration of DHP1A obviously reduced the levels of alanine aminotransferase, aspartate aminotransferase, lactate dehydrogenase and 8-hydroxy-2′-deoxyguanosine in the serum of mice treated with carbon tetrachloride (CCl_4_), suggesting the hepatoprotective potential of this polysaccharide. Moreover, DHP1A decreased the expressions of tumor necrosis factor-α, interleukin-1β, monocyte chemoattractant protein-1, macrophage inflammatory protein-2, CD68 and phosphorylated I*κ*Bα (p-I*κ*Bα) in the CCl_4_-treated mice. These results revealed that the hepatoprotective effect of DHP1A was partly attributed to its anti-inflammatory action.

## Introduction

*Dendrobium huoshanense*, a perennial orchid species, has been used as a folk medicine in China for centuries. Its stems possess variety of restorative and therapeutic functions, which has been exploited as a valuable beverage, named as ‘ShiHu’. Pharmacological research works indicate that polysaccharides are the major active components in *D. huoshanense*. Through *in vitro* assays, it was reported that the polysaccharides from *D. huoshanense* were capable of activating immune cells,[1] scavenging free radicals [2] and inhibiting glycation.[3] Moreover, the *in vivo* assays have demonstrated that oral administration of *D. huoshanense* polysaccharides can attenuate the streptozotocin-induced cataract [4] and modulate the immune responses in intestines, spleen and liver.[5]

Carbon tetrachloride (CCl_4_), a potent hepatotoxic chemical, can rapidly cause an oxidative stress and inflammatory response to hepatocytes, which leads to an acute hepatic necrosis. Excessive production of free radicals (including ·CCl_3_ and ·OOCCl_3_) originating from the cytochrome P450 metabolism of CCl_4_ is responsible for the initiation of liver injury, and the consequent inflammatory response that further aggravates the extent of injury.[6] Some studies suggest that the activation of intrahepatic macrophages (i.e. Kupffer cells) plays a key role in the CCl_4_-induced inflammatory response via release of various cytokines and chemokines, such as tumor necrosis factor (TNF)-α, interleukin (IL)-1, IL-6, monocyte chemotactic protein (MCP)-1 and macrophage inflammatory protein-2 (MIP-2).[7,8] Besides, the nuclear factor-kappa B (NF-*κ*B) is believed to be associated with the hepatic inflammatory injury induced by CCl_4_.[9]

In recent years, natural polysaccharides have been reported to be capable of enhancing liver resistance against CCl_4_ toxicity.[10–12] Huang et al. [13] previously reported that the crude polysaccharides from *D. huoshanense* could protect the liver from CCl_4_ hepatotoxicity, but the constituents in the crude *D. huoshanense* polysaccharides were so complex that the relationship between the hepatoprotection and polysaccharides still remained obscure. Moreover, many views considered that the protective effects of polysaccharides on CCl_4_-induced liver injury were mainly due to the antioxidative properties of the polysaccharides, but investigations concerning the mechanisms of inflammatory regulation facilitated by polysaccharides were relatively limited. In a previous work, a homogenous polysaccharide (named as DHP1A) was obtained from *D. huoshanense* via a water extraction–ethanol precipitation, anion exchange and size exclusion chromatography, and its structural characterization was performed by the various spectral methods.[14] In this study, we further assessed the protective effects of DHP1A on CCl_4_-induced liver injury, and proposed that the inhibition of inflammatory responses may be an important hepatoprotective mechanism of this polysaccharide.

## Materials and methods

### Materials

*D. huoshanense* was collected and propagated in our lab under the conditions as previously described.[15] DEAE-cellulose D-52 and Sephadex G-100 were purchased from Amersham Pharmacia Biotech (London, England) and Sigma-Aldrich Co. (St. Louis, MO, USA), respectively. Enzyme linked immunosorbent assay (ELISA) kits for detection of TNF-α, IL-1β, IL-10 and 8-hydroxy-2′-deoxyguanosine (8-OHdG), were purchased from R&D Systems China Co., Ltd. (Shanghai, China). The primary antibodies against CD68 and phosphorylated IκBα (p-IκBα) (Ser 32) were purchased from Biorbyt (Cambridgeshire, United Kingdom) and Santa Cruz (CA, USA), respectively. TRIzol® Reagent was purchased from Invitrogen (Carlsbad, USA). iScript™ cDNA Synthesis kit and iTaq™ Universal SYBR® Green Supermix kit were purchased from BIO-RAD (CA, USA). Histostain™-Plus Kits and DAB kit were purchased from ZSGB-Bio (Beijing, China).

### Animals

Male Kunming mice (SPF grade, 23 ± 2 g) were obtained from the Experimental Animal Center of Anhui Medical University, China. The mice were housed in an air-conditioned room (25 ± 2 °C) with a normal light/night cycle. The animal care and experimental protocols complied well with the national guidelines for the care and use of animals.

### Preparation of DHP1A

The preparation of DHP1A from crude polysaccharides of *D. huoshanense* was carried out according to previous methods.[14] DHP1A is proved to be a homogenous polysaccharide fraction with a molecular weight of 6700 Da and consists of mannose, glucose and galactose in the molar ratio of 2.5:16.0:1.0, which was evidenced by high performance liquid chromatography, gas chromatography-mass spectrometer and nuclear magnetic resonance. The carbohydrate concentration of DHP1A was determined to be 98.9% using phenol–sulphuric acid method,[16] while proteins were not detected.

### Experimental design

After acclimatized for 7 days, 80 mice were randomly divided into eight groups (*n* = 10 per group), including (1) blank control group (distilled water); (2) silymarin treatment group (25 mg/kg body weight (BW)); (3) low-dose DHP1A treatment group (100 mg/kg BW); (4) high-dose DHP1A treatment group (200 mg/kg BW); (5) CCl_4_ group (0.2% CCl_4_ dissolved in olive oil, 10 mL/kg BW); (6) silymarin (25 mg/kg BW) + CCl_4_ treatment group; (7) DHP1A (100 mg/kg BW) + CCl_4_ treatment group and (8) DHP1A (200 mg/kg BW) + CCl_4_ treatment group. DHP1A or silymarin was dissolved in distilled water and orally administered to the mice at the dose of 10 mL/kg BW once daily for 14 days. The control group (1) and CCl_4_ group (5) were orally administered with distilled water at the same dose. After 4 hours of the last treatment, the mice (groups 5–8) were intraperitoneally injected with 0.2% CCl_4_ solution at the dose of 10 mL/kg BW, while the rest mice (groups 1–4) were treated with olive oil at the same dose. Twenty‑four hours later, the blood was collected from the inner canthus cave and centrifuged at 3000 rpm for 10 min to separate the serum, which was stored at −70 °C until analysis. Subsequently, the mice were sacrificed by cervical dislocation and their livers were quickly removed. The left lobe of the liver was fixed by immersing in 10% formalin solution, while the rest of the liver was quickly frozen with liquid nitrogen and stored at −70 °C until analysis.

### Assessment of liver function

In order to evaluate the degree of liver injury, the levels of alanine aminotransferase (ALT), aspartate aminotransferase (AST), lactate dehydrogenase (LDH) and 8-OHdG in the serum were measured by commercially available kits.

### Histopathological analysis

The hepatic tissue fixed with formalin solution was embedded in paraffin, sliced into sections of 5 μm thickness and stained with hematoxylin and eosin (H&E). The pathological changes of the liver tissues were examined using an optical microscope (Nikon, Japan) at standard magnification.

### Cytokine assays

The levels of TNF-α, IL-β and IL-10 in the serum were measured by ELISA kits according to the manufacturer's instruction.

### Real-time quantitative polymerase chain reaction

Total RNA was extracted from the hepatic tissue by using TRIzol reagent and reverse transcribed into first cDNA strand with iScript™ cDNA Synthesis Kit.

The mRNA expression levels of monocyte chemoattractant protein-1 (MCP-1), MIP-2 and glyceraldehydes-3-phosphate dehydrogenase (GAPDH) were determined by real-time quantitative polymerase chain reaction (qPCR). The primer sequences obtained from Sangon Biotech limited Co. (Shanghai, China) were listed as follows: 5′-TCACCTGCTGCTACT CATTCAC-3′ and 3′-CCATTCCTTCTTGGGGTCAG-5′ for MCP-1(NM-011333.3); 5′-CAGAAGTCATAGCCACTCTCAAG-3′ and 3′-TCCTCCTTTCCAGGTCAGTTAG-5′ for MIP-2(NM-009140.2); and 5′-AGGCCGGTGCTGAGTATGTC-3′ and 3′-TGCCTGCTTCACCACCTTCT-5′ for GAPDH (M32599). The PCR reactions were carried out by using iTaq™ Universal SYBR® Green Supermix kit in a real-time fluorescent quantitative PCR instrument (iQ5, BIO-RAD, Hercules, USA). GAPDH was used as an internal reference, and the relative quantification of objective genes was calculated according to the 2^−ΔΔCt^ method.

### Immunohistochemical assay

The expression of CD68 in the livers was investigated by the immunohistochemical staining according to the manufacturer's procedure. In brief, liver tissue sections (4 μm thick) after deparaffinized and heated in boiling sodium citrate buffer solution (0.01 M, pH 6.0) for 5 min were blocked with 3% H_2_O_2_ and 10% goat serum, successively and then incubated with a polyclonal rabbit anti-CD68 antibody (1:100 dilution) at 4 °C overnight. After washed thrice with phosphate buffer solution (PBS), the sections were incubated with goat anti-rabbit IgG (37 °C, 30 min) and S-A/HRP (37 °C, 30 min), successively. At last, the sections were stained with DAB solution and hematoxylin, and viewed using a Nikon 80i microscope (Tokyo, Japan).

### Western blot analysis

The western blot technique was used to analyse the expression of p-IκBα in the cytoplasm of hepatocytes according to previous protocols.[17] In short, proteins extracted from the liver tissue were separated by 10% SDS-polyacrylamide gel electrophoresis and transferred to the nitrocellulose membrane. After blocked with 5.0% skim milk, the membrane was incubated with p-IκBα primary antibody overnight at 4 °C, horseradish peroxidase-conjugated secondary anti-body and chemiluminescence reagents successively. After that, the membrane was investigated by Gel Doc™ XR^+^ and ChemiDOC™ XRS^+^ gel documentation systems (BIO-RAD, Hercules, USA). As an internal reference, α-tubulin was used in this assay.

### Statistical analysis

Data were expressed as mean ± standard deviation. The statistical difference between groups was determined by one-way analysis of variance followed by the Tukey's test. Difference was considered significant when *p* < 0.05.

## Result and discussion

It is well known that natural polysaccharides are a type of active biomacromolecules that can modulate the innate immunity and adaptive immunity of body through reacting with diverse immune cells.[18,19] Some of them have exhibited a variety of beneficial preventive and therapeutic properties, which attract more and more attention.[20] In this study, we demonstrated that DHP1A, a polysaccharide from *D. huoshanense*, could effectively enhance the liver resistance against CCl_4_-induced inflammatory injury.

**Figure 1.**
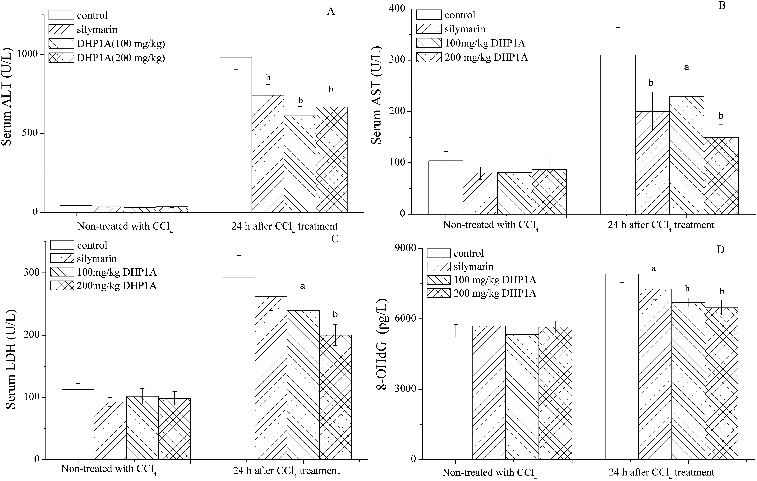
Effects of DHP1A on CCl_4_-induced liver injury. (A) ALT; (B) AST; (C) LDH; (D) 8-OHdG. ^a^ *p* < 0.05 and ^b^ *p* < 0.01 as compared with the CCl_4_ treatment group.

CCl_4_-induced liver injury is a good model to evaluate hepatoprotective agents. Following the CCl_4_ injection, the levels of ALT, AST and LDH in the serum of mice were increased to 2286%, 298% and 261% of the control, respectively. Moreover, the production of 8-OHdG, an oxidative product of DNA,[21] was increased to 144% of the control. These results showed that CCl_4_ toxicity not only affected cell membranes but also cell nuclei. DHP1A pretreatment attenuated the increase of these biochemical parameters caused by CCl_4_, suggesting the obvious hepatoprotective effect of this polysaccharide. When the mice were pretreated with DHP1A at 200 mg/kg BW, the levels of ALT, AST, LDH and 8-OHdG following the CCl_4_ treatment were 1551%, 155%, 178% and 118% of the corresponding control, respectively, which were significantly lower than these in the mice treated with CCl_4_ alone (Figure 1). Furthermore, the DHP1A treatment alone was proven to be of low toxicity. Silymarin, a hepatoprotective drug,[22] was used as a positive control, which exhibited similar results.

**Figure 2.**
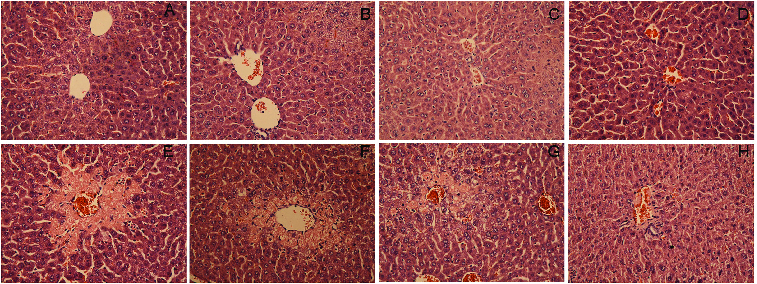
Pathological changes of liver tissues in each treatment group. H&E staining of liver section from (A) blank control group; (B) 25 mg/kg silymarin treatment group; (C) 100 mg/kg DHP1A treatment group; (D) 200 mg/kg DHP1A treatment group; (E) CCl_4_ treatment group; (F) 25 mg/kg silymarin + CCl_4_ treatment group; (G) 100 mg/kg DHP1A + CCl_4_ treatment group and (H) 200 mg/kg DHP1A + CCl_4_ treatment group.

The results were further confirmed by the investigations of pathological liver tissue sections. As shown at Figure 2, the pretreatment of DHP1A (Figure 2(G) and 2(H)) significantly reduced the centrilobular necrosis induced by CCl_4_ as compared with the mice treated with CCl_4_ alone (Figure 2(E)). Moreover, it was easy to find little difference between the normal mice and the mice pretreated with DHP1A or silymarin alone, indicating the low toxicity of DHP1A and silymarin (Figure 2(A)–(D)).

**Figure 3.**
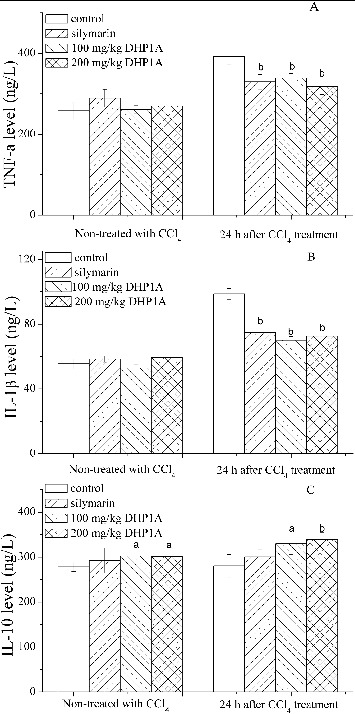
Effects of DHP1A on cytokines expressions. (A) TNF-α; (B) IL-1β; (C) IL-10. ^a^ *p* < 0.05 and ^b^ *p* < 0.01 as compared with the CCl_4_ treatment group; *p < 0.05 as compared with the blank control group.

Most of liver diseases are accompanied by the inflammatory responses that stem from a systemic defence action against various tissue damages, but failure to appropriately regulate the self-response would result in serious pathological consequences.[23] In the early phase of CCl_4_ hepatotoxicity, TNF-α and IL-1β have been regarded as the primary pro-inflammatory cytokines originating from activated Kupffer cells to initiate the inflammatory cascade.[10,24] As shown in Figure 3, the levels of TNF-α and IL-1β in the mice treated with CCl_4_ alone were increased to 152% and 177% of the corresponding control, respectively. However, DHP1A pretreatment alleviated the changes of the two pro-inflammatory cytokines. IL-10 as an anti-inflammatory cytokine can disturb the synthesis of pro-inflammatory cytokines.[11] Following the DHP1A treatment, the IL-10 production significantly increased in the mice treated or non-treated with CCl_4_, possibly suggesting that IL-10 played a key role in modulating the inflammatory response caused by DHP1A.

**Figure 4.**
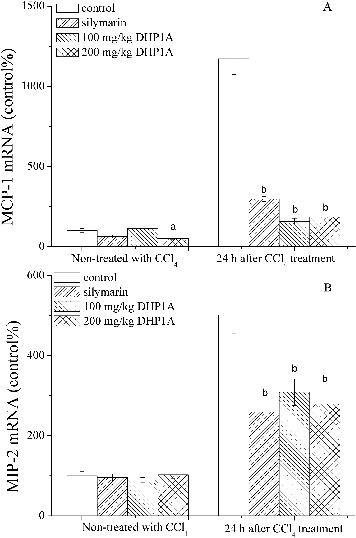
Effects of DHP1A on chemokine mRNA expression. Expressive levels of (A) MCP-1 mRNA and (B) MIP-2 mRNA were measured in the livers of different treatment groups. GAPDH was used as internal controls. ^a^ *p* < 0.05 and ^b^ *p* < 0.01 as compared with the CCl_4_ group; *p < 0.05 as compared with the blank control group.

**Figure 5.**
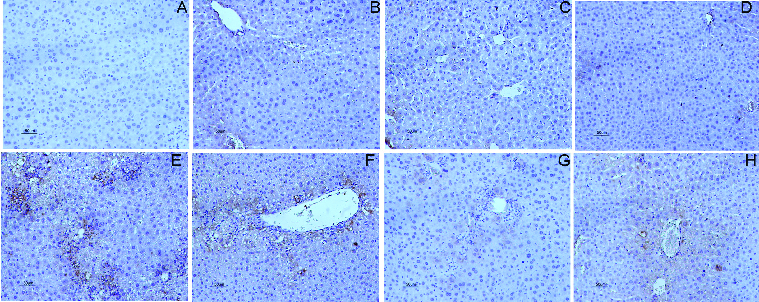
Immunostaining of CD68 in the liver section. (A) blank control group; (B) 25 mg/kg silymarin treatment group; (C) 100 mg/kg DHP1A treatment group; (D) 200 mg/kg DHP1A treatment group; (E) CCl_4_ treatment group; (F) 25 mg/kg silymarin + CCl_4_ treatment group; (G) 100 mg/kg DHP1A + CCl_4_ treatment group and (H) 200 mg/kg DHP1A + CCl_4_ treatment group.

TNF-α is a pleiotropic cytokine that regulate the expressions of certain chemokines and induce the recruitment of leukocytes into damaged sites.[25] MCP-1 and MIP-2 have been reported to be typical pro-inflammatory chemokines for the recruitments of neutrophil and mononuclear leukocytes, and exert important impact on the inflammatory injuries of livers.[26,27] CD68 is a glycoprotein expressed in mononuclear phagocyte lineage cells, including monocytes, Kupffer cells and most macrophages.[28] Following the CCl_4_ treatment alone, the mRNA expression of MCP-1 increased to 1170% of that in the normal mice, while the mRNA expression of MIP-2 increased to 500% of that in the normal mice (Figure 4). Meanwhile, the increased CD68 positive cells in liver tissues were obviously observed in the mice treated by CCl_4_ alone, indicating the occurrence of inflammatory response (Figure 5(E)). As compared with the mice in the model group, the mice pretreated with DHP1A exhibited a significant resistance against the expressions of these inflammatory mediators.

**Figure 6.**
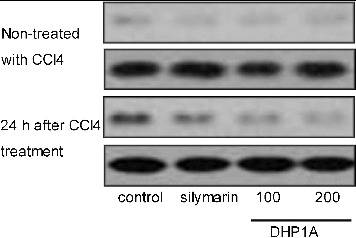
Effects of DHP1A on p-I*κ* B expression.

Besides, increasing evidence shows that the activation of NF-*κ*B is an important mechanism involved in the CCl_4_-induced hepatotoxicity.[2,24] In normal state, NF-*κ*B is combined with its inhibitory protein (I*κ*B) and exists in the cytoplasm without activity. Once cells are subjected to pathological stimuli, NF-*κ*B shifts to the nucleus following the phosphorylation and degradation of I*κ*B, and regulates inflammatory gene expression.[9] Our results indicated that DHP1A could attenuate the phosphorylation of I*κ*B caused by CCl_4_ (Figure 6), which possibly inhibited the activation of NF-*κ*B pathway.

## Conclusions

In summary, DHP1A exhibited obvious abilities to inhibit the inflammatory response caused by CCl_4_ via decreasing the expressions of inflammatory cytokines, chemokines, CD68 and p-I*κ*Bα, suggesting the hepatoprotective potential of this polysaccharide. Moreover, the low hepatoxicity of DHP1A would provide the foundation for itself as a safe food supplement for the prevention of livers disease development.
